# P-Body Components, microRNA Regulation, and Synaptic Plasticity

**DOI:** 10.1100/tsw.2007.206

**Published:** 2007-11-02

**Authors:** Jens Hillebrand, Scott A. Barbee, Mani Ramaswami

**Affiliations:** ^1^ Smurfit Institute of Genetics and TCIN, Lloyd Building, Trinity College Dublin, Dublin-2, Ireland; ^2^ Department of Molecular and Cellular Biology, University of Arizona, Tucson, AZ 85721, USA; ^3^ ARL Division of Neurobiology, University of Arizona, Tucson, AZ 85721, USA; ^4^ Department of Biological Sciences, University of Denver, Denver, CO 80208, USA

**Keywords:** P-body, microRNA, synaptic plasticity, neuronal granules, translational control, Me31B, FMRP, Drosophila

## Abstract

What is the protein apparatus required for microRNA (miRNA) function and translational repression in neurons? This article reviews our recent work on Me31B, a conserved P-body protein present on Staufen-containing neuronal and maternal ribonucleoprotein (RNP) particles, which is required for dendrite morphogenesis and miRNA function *in vivo*. In addition, it provides new data to show that Me31B is present on and regulates formation of P-bodies in the *Drosophila* wing disc, where it has a general role in the regulation of miRNA function. While illuminating the function of this important RNA regulatory molecule, it also brings into focus a hypothesis of potentially broad significance. Namely, that P-body proteins may play important roles in regulation of dendrite-localized mRNAs and, thereby, in synaptic plasticity. A wide range of protein localization and early functional data support this hypothesis. We also discuss current knowledge of RNP particles that mediate translational repression and the implications of these findings for understanding translational control in neurons.

## INTRODUCTION

A significant body of work argues strongly for the role of new protein synthesis in the establishment of long-term memory (LTM). For example, studies of long-term potentiation (LTP) and long-term depression (LTD) in mammals have identified two distinct phases of plasticity: an early phase (typically lasting 1–3 h) that is independent of new protein synthesis and a late phase (lasting >8 h) that is dependent on new protein synthesis[1]. Many recent investigations of molecular mechanisms underlying new protein synthesis have focused on local regulation of mRNA translation. In neurons, local mRNA translation serves many important functions during development or growth of axons and dendrites, and the regulation of synaptic plasticity. Local mRNA translation is regulated by sequence motifs within mRNAs in combination with specific RNA binding proteins and noncoding RNAs (ncRNAs). Together, the composition of these ribonucleoprotein (RNP) complexes determines whether mRNAs are transported to a specific location (e.g., to the synapse), locally translated, or targeted for storage and/or degradation.

Many excellent reviews have been written covering the topics of mRNA transport[2,3,4], local translation[1,5,6], RNP granule structure and function[7,8,9], and ncRNA function[10,11]) in neurons. Readers are directed to these articles for more detailed discussion of each of these topics.

Here we focus on recent evidence suggesting that neuronal RNPs, particularly in *Drosophila* neurons, are similar in structure and function to cytoplasmic RNA processing bodies (also called GW- or DCP-bodies, but hereafter referred to as P-bodies) – conserved RNPs present in all cells from yeast to mammals[12]. P-bodies, initially identified as cytoplasmic sites for mRNA turnover to which RNA decapping and degradative enzymes are localized, have since been shown to also act as sites for transient storage of translationally repressed mRNAs[13]. Additionally, recent evidence suggests that there is a link between P-bodies and the function of small interfering RNAs (siRNAs) and microRNAs (miRNAs)[8,14,15,16].

Translational regulatory proteins that provide local control of mRNA translation in neurons are beginning to be identified. These proteins include not only classical translational repressor/activator proteins, but also molecules proposed to function in the miRNA pathway (such as the Fragile X Mental Retardation Protein, FMRP). Given the demonstrated association of several P-body proteins with FMRP- and FMRP/Staufen-containing RNPs, it is of obvious interest to ask whether P-body proteins are involved in the function of neuronal miRNAs. It has recently been shown that specific miRNAs and components of the miRNA pathway regulate important aspects of neuronal development and plasticity[17,18]. Below, we discuss in greater detail the implications of P-body structure and function on miRNA-mediated regulation of neuronal development, synaptic plasticity, and long-term memory. Specifically, do P-bodies and/or P-body proteins play a role in the mechanism underlying local protein synthesis at specific synapses?

## NEURONS CONTAIN A DIVERSE POPULATION OF RNPS WITH CONSERVED STRUCTURE AND FUNCTION

Regulation of local mRNA translation in neurons is mediated both during transport along neuronal processes and at synaptic sites. During transport, translational repression occurs in large RNPs broadly termed "neuronal granules" or "transport granules" (which we hereafter refer to as NGs). At least two excellent reviews recently categorized macroscopic (visible by light microscopy) NGs into a number of distinct RNP "subclasses" based on homology to non-neuronal cytoplasmic RNPs[7,9]. These granule "subclasses" include P-bodies, stress granules, the RNA-induced silencing complex (RISC), RNA granules, and transport particles[9]. Each is characterized by the presence (or absence) of specific protein components. This classification scheme is supported, in part, by the observation that *Drosophila* neurons do not appear to contain a homogenous population of RNPs[12].

However, at least two lines of evidence suggest that most NGs are, in fact, structurally and functionally related. First, NGs in cultured *Drosophila* neurons contain conserved components of mammalian NGs and other cytoplasmic RNPs (Table 1). For example, most NGs can be broadly identified by the presence of three highly conserved proteins: the dsRNA-binding protein, Staufen[19,20,21]; the extensively studied FMRP[12,22,23]; and the DEAD-box RNA helicase, RCK/Me31B/Dhh1p[24,25,26]. Second, all NGs are sites for the storage of translationally repressed mRNAs[27,28,29,30]. The existence of biochemically diverse particles is at least partially accounted for by observations indicating a rapid exchange of proteins between these particles, or during the maturation of particles as they grow in size. For example, fluorescence recovery after photobleaching (FRAP) analysis reveals that many specific RNP-associated proteins rapidly shuttle in and out of NGs as well as RNPs in non-neuronal cells[12,31,32,33,34]. At present, data are most consistent with a model in which RNPs are highly dynamic, and suggest that NGs are cellular compartments where mRNAs are remodeled and sorted for mRNA decay, translational repression, and/or mRNA storage (Fig. 1). Taken together with our recent finding that at least ten P-body proteins are present on Staufen- and FMRP-containing NGs in *Drosophila* neurons[12], these data suggest that NGs share a fundamentally conserved structure and function with cytoplasmic P-bodies.

**TABLE 1**
**Conserved Components of P-Bodies and NGs**

| Protein Class | Mammalian, Fly, and Yeast P-Bodies[a] | *Drosophila* NGs[b] | Mammalian NGs[c] |
|---|---|---|---|
| Conserved NG components | | | |
| Fragile X-like | FMRP | dFMR1 | FMRP, FXR1, FXR2 |
| RNA transport | Stau | Stau, Btz | Stau, Btz |
| DEAD-box RNA helicase | RCK/Me31B/Dhh1p | Me31B | ? |
| Core P-body components | | | |
| 5’ to 3’ RNA exoribonuclease | Xrn1p | Pcm | ? |
| Sm-like and FDF domain; involved in repression | RAP55/Tral/Scd6p[i] | Tral | ? |
| Enhancers of decapping | Edc3p, Pat1p[h] | ? | ? |
| Sm-like involved in decapping | Lsm1-7p | ? | ? |
| Decapping enzymes | Dcp1p/Dcp2p | DCP1 (DCP2 not examined) | |
| miRNA/siRNA (RISC) components | | | |
| miRNA, siRNA machinery | mAGO1, mAGO2 | Ago-2 | ? |
| NMD components | | | |
| Helicase required for NMD[g] | Upf-1 | Upf-1 | ? |
| RNA-binding proteins and translational repressors | | | |
| Cap-binding | eIF4E[d, j] | eIF4E[also f*] | Under some conditions |
| eIF4E-binding | eIF4E-T[j] | Cup | ? |
| Poly(A)-binding | PABP[d] | PABP[f*] | ? |
| Zip-code binding | ? | Imp | ZBP1 |
| Puf domain | ? | Pum | ? |
| CCHC Zn-finger domain | ? | Nanos[also e*] | ? |
| Y-box | ? | Yps | ? |

? Ortholog present, but association with RNA granules has not been described.

[a] [19,23,25,31,37,76]

[b] [12](except PABP)

[c] [20,24,32,77]

[d] eIF4E and PABP can associate with P-bodies in yeast under conditions of translational arrest induced by glucose deprivation[78].

[e] [79]*

[f] [80]* (*Refs. e and f describe particles likely to be, but not clearly established as, neuronal RNA granules.)

[g] UPF1 has also been shown to interact with Stau in a Stau-mediated NMD pathway[81].

[h] Pat1p acts as a general repressor of translation in yeast[40].

[I] [12,82]

[j] [31]

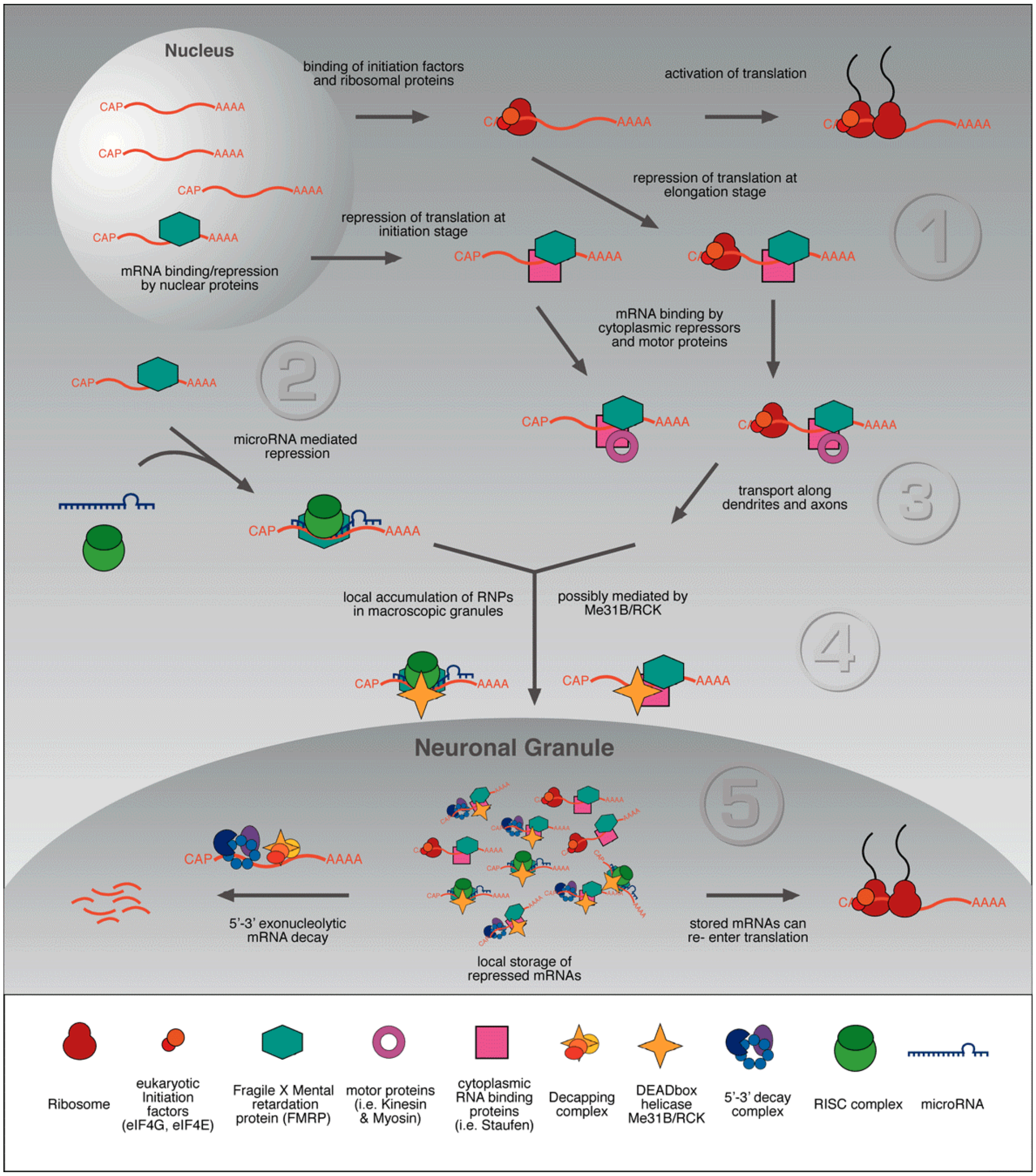

**FIGURE 1**. Model. Local protein synthesis in neurons plays a major role in neuronal development and synaptic plasticity. In order to regulate local translation, a neuron uses different mechanisms to repress, transport, store, degrade, and derepress mRNAs. This model gives an overview of different mRNP particles and their interaction. (1) Translation is initiated by the binding of eukaryotic initiation factors (eIFs) and ribosomal proteins to the mRNA. At this stage, specific mRNA binding proteins can repress translation at the elongation stage. Alternatively, mRNAs can be repressed before ribosomal proteins bind to the mRNA, blocking translation at the initiation stage. mRNPs containing mRNAs blocked at those two stages can be distinguished by the presence or absence of ribosomes. (2) The miRNA pathway represents an alternative way to repress mRNA translation. miRNAs are noncoding small RNAs (on average 22 nt long) derived from double-stranded pre-miRNAs. When attached to the RISC, miRNAs bind to specific sequences in the UTR of their target mRNAs and repress translation. As many miRNAs are expressed in the nervous system and a role for miRNAs in synaptic plasticity and memory formation has been shown, a major role for miRNA-mediated translational repression in neurons is implicated. (3) Translationally repressed mRNAs are bound by specific RNA binding proteins, translational repressors, and motor proteins, and form diverse mRNPs, which are transported along dendrites and axons. (4) Locally, i.e., at synapses or dendritic branch points, diverse mRNPs accumulate in macroscopic NGs. A main component of NGs is the DEAD-box helicase Me31B/Rck, which is required for the formation of somatic P-bodies; thus implicating a role for Me31B/RCK in the formation of macroscopic NGs. (5) NGs are highly dynamic structures. FRAP experiments in neurons showed that proteins and mRNAs can enter and exit mRNP particles very rapidly. Furthermore, recent data suggest that NGs are cellular compartments where mRNAs are remodeled and sorted. mRNAs can either be repressed and stored locally, enter the decay pathway (proteins involved in 5'-3' exonucleolytic decay are present in NGs), or mRNAs can exit NGs and re-enter translation, resulting in local protein synthesis.

The observation that NGs and P-bodies are structurally and functionally related is potentially important for two reasons. First, it would allow for the identification of a large number of well-characterized P-body proteins as regulators of neuronal translational control. Second, it poses the interesting possibility that local dendritic pools of mRNA may be regulated by an additional mechanism – local mRNA turnover. Together, this raises an important question that needs to be addressed. What neuronal processes and mechanisms are regulated by P-bodies?

## P-BODY COMPONENTS ARE REGULATORS OF MRNA DECAY AND GENERAL TRANSLATIONAL REPRESSION

P-bodies are large, macroscopic, cytoplasmic aggregates of translationally repressed mRNAs associated with the translational repression and decay machinery[8,14]. P-bodies were initially thought to be primarily sites of mRNA decapping and 5' to 3' exonucleolytic degradation[25]. Core P-body protein components are conserved from yeast to mammals (Table 1) and include: (a) the mRNA decay machinery, including the decapping enzymes Dcp1p/Dcp2p and the 5' to 3' exoribonuclease, Xrn1/Pcm; (b) the activators of decapping, Edc3p, RAP55/Scd6p, and the Lsm1p-7p complex; and (c) the enhancers of decapping and general translational repressors, Me31B/RCK/Dhh1p and Pat1p[8]. P-bodies also contain components of the RISC, including the Argonaute proteins (Ago1 and Ago2), indicating a role in miRNA/siRNA-mediated gene silencing[35,36,37,38].

P-bodies are now thought to also be sites of translational regulation via miRNA, nonsense-mediated decay (NMD), and general repression pathways[8]. Several lines of evidence suggest that P-body proteins also have direct functions in general translational repression in higher eukaryotes. First, the *Xenopus* ortholog of yeast Scd6p (RAP55) represses translation of reporter mRNAs in cell extract[39]. Second, the decapping coactivators, Pat1p and Dhh1p, are general repressors of general translation in yeast[40]. Finally, orthologs of Dhh1p (Xp54, CGH-1, and Me31B) function in the translational repression of specific maternal mRNAs in *Xenopus, C. elegans,* and *Drosophila,* respectively[41,42,43,44].

The analysis of P-bodies in yeast and mammalian cells is identifying critical protein interactions with P-bodies and specific protein functional domains that mediate interactions essential for P-body formation, translational repression, and mRNA decay. These analyses will provide a wealth of tools to test the importance of specific proteins, protein assemblies, protein interactions, and cell biological processes in neuronal development, function, and plasticity.

## MIRNAS AND SYNAPTIC PLASTICITY

Computational analysis of the human genome suggests that nearly one-third of human genes are regulated by ~500 miRNAs, a discovery of profound significance for gene regulation in multicellular organisms[45]. Of these miRNAs, a large number are expressed in the mammalian nervous system[30,46,47], and have been shown to regulate neuronal mRNAs directly[30,48,49].

Three important observations indicate a role for the miRNA pathway in synaptic plasticity and LTM formation. First, it has recently been shown that in the rat hippocampus, the brain-specific miRNA, miR-134, controls dendritic spine morphogenesis via regulation of a mRNA encoding LimK1, a protein kinase that has been implicated in spine development[18]. In mammals, dendritic spine morphogenesis is thought to correlate strongly with synaptic plasticity[50,51,52]. Second, the FMRP protein has been shown to be required for dendritic spine morphogenesis in humans and synaptic plasticity in *Drosophila*[53,54,55]. Defects in FMRP results in Fragile X Syndrome, the most common form of inherited mental retardation in humans[6]. FMRP has been shown to interact with components of the miRNA pathway and, in some cases, is thought to be part of the RISC complex[56,57,58]. Finally, recent results in *Drosophila* directly connect the RISC machinery with synaptic plasticity and LTM formation. In the olfactory system, Ashraf et al.[17] showed that the RISC component Armitage is required for formation of LTM. Moreover, Armitage and Dicer-2 are involved in the regulation of synaptic translation of ∝-calcium/calmodulin–dependent protein kinase II (∝-CaMKII), a predicted target of the miRNAs miR-280 and miR-289. The neuronal RNA binding proteins Staufen and Kinesin Heavy Chain are also predicted miR-280 targets, whose neuronal expression is similarly enhanced in Dicer-2 and *armitage* mutants. Interestingly, LTM formation correlated strongly with increased ∝-CaMKII synthesis via an Armitage- or miRNA-dependant mechanism. These and other related studies of miRNAs in neurons have been recently reviewed[10,59,60].

## P-BODY COMPONENTS REGULATE MIRNA FUNCTION

The first indication of a link between P-bodies and the miRNA/siRNA machinery was the discovery that the mammalian Argonaute proteins, components of the RISC complex, colocalized to cytoplasmic foci with the P-body proteins Dcp1/Dcp2[37,38,61]. Six additional lines of evidence indicate a relationship between miRNA function and P-bodies. First, depletion of GW182 (a mammalian P-body component – hence the name GW bodies) in human or *Drosophila* cells impairs silencing by miRNAs (and to a lesser extent) siRNAs[35,37,62,63,64]. Second, AIN-1, a *C. elegans* protein similar to GW182, was shown to interact with components of the RISC complex and is required to target the Argonaute protein to cytoplasmic P-bodies[36]. Third, human and *Drosophila* cells depleted of the Dcp1/Dcp2 complex have impaired miRNA function[35,37,65]. Fourth, Me31B and RCK (the mammalian ortholog of Dhh1p) have been shown to regulate miRNA function in both *Drosophila* and mammalian cells[12,62]. Fifth, miR16 is required for AU-rich element (ARE)–mediated mRNA degradation, a process that requires an ARE binding protein (tristertraprolin or TTP) shown to localize to mammalian P-bodies[34,66]. Finally, reporter mRNAs targeted for translational repression by miRNAs become targeted to mammalian P-bodies in a miRNA-dependant manner[67]. Two recent studies have shown that the formation of macroscopic *Drosophila* and mammalian P-bodies are not required for miRNA-mediated gene regulation per se[19,68]. This leads to a model in which, although P-body components play essential roles in miRNA function, aggregation of proteins into large P-bodies is a consequence of, rather than a requirement for, miRNA activity.

## *DROSOPHILA* ME31B IS REQUIRED FOR P-BODY FORMATION AND MIRNA FUNCTION

Our recent published work shows that Me31B, a member of the RCK/Dhh1p family of DEAD-box RNA helicases, localizes to NGs in cultured *Drosophila* neurons[12]. In yeast and *Drosophila* S2 cells, it is required for the formation of P-bodies[19,25]. Observations in cultured cells and subsequent *in vivo* studies implicate Me31B in general and miRNA/siRNA-mediated pathways for mRNA silencing[12,40,62]. Consistent with a role for Me31B in repression of dendritic branching, a process likely to be regulated by local translational control mechanisms, Me31B as well as two physically associated proteins, FMRP and Trailerhitch (Tral, the *Drosophila* ortholog of RAP55/Scd6p), have been shown to be required for dendrite morphogenesis in *Drosophila* sensory neurons[12].

Here we present some important additional data that support a role for Me31B in P-body formation and miRNA function *in vivo*. Consistent with earlier observations in *Drosophila* S2 cells[19], Fig. 2 shows that Me31B localizes to endogenous P-bodies in *Drosophila* wing imaginal disc cells. In accordance with previous observations in yeast[25], Me31B/RCK/Dhh1p is required for macroscopic P-body formation in wing discs. Thus, depletion of Me31B either in *me31b* mutant cell clones created by mitotic recombination using the FRT/FLP system, or by RNA interference (RNAi), results in the loss of macroscopic P-bodies visualized by staining for independent P-body marker proteins. Me31B-deficient cells show greatly reduced number of bright foci that contain the core P-body components, Dcp1 (Fig. 2A–C) and Pcm (Fig. 2D–F).

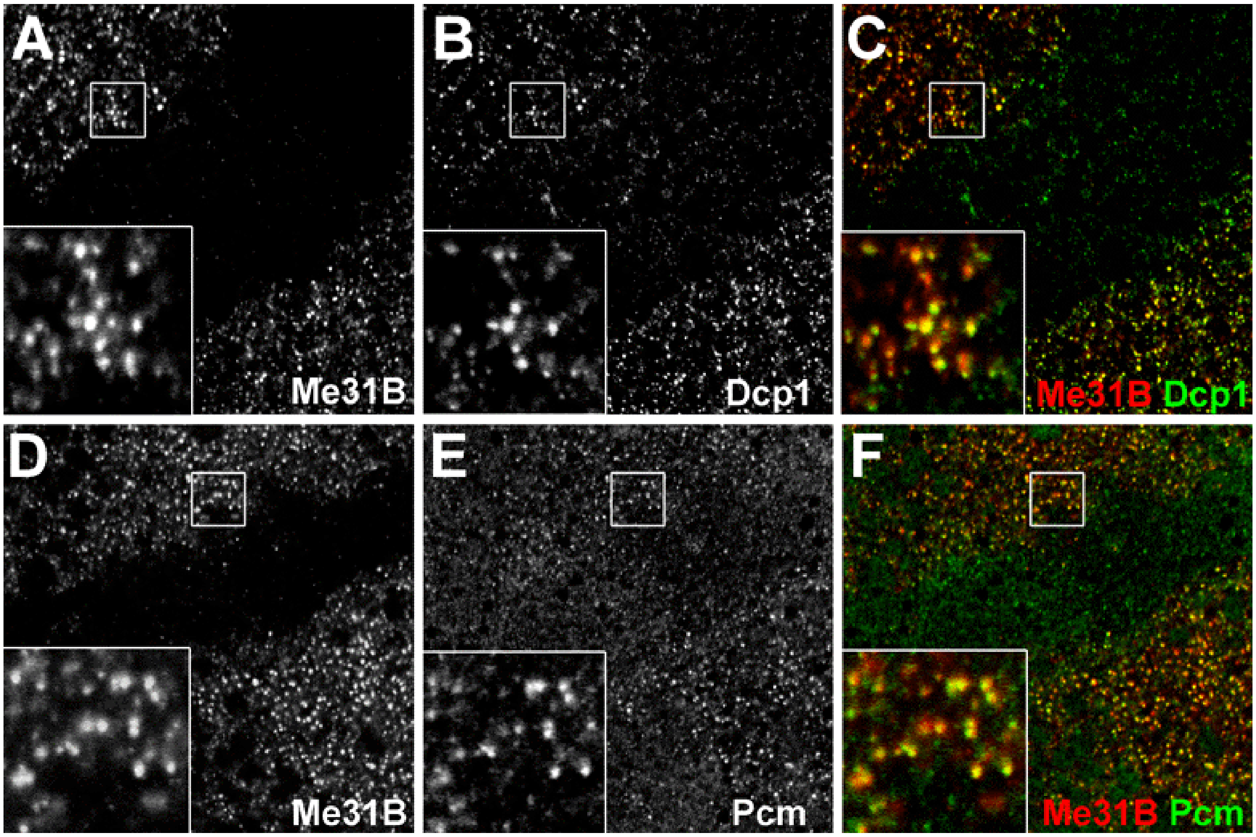

**FIGURE 2**. Depletion of Me31B reduces Dcp1 and Pacman (Pcm) expression. High-resolution images of wing imaginal discs, showing the colocalization of Me31B in cytoplasmic foci with the P-body components Dcp1(B and C) and Pcm (E and F), the *Drosophila* homologue of the exoribonuclease Xrn1. Depleting Me31B by RNAi[12] induces the loss of P-bodies in the wing imaginal disc; less bright Dcp1 and Pcm expressing foci can be observed in areas where Me31B is depleted (marked by the absence of Me31B in A). Expression of a Me31B hairpin construct[12] was driven with *patched*-Gal4 (Bloomington), reducing Me31B expression at the anterior/posterior boundary of the wing imaginal disc. Wing discs of 3rd instar larvae were dissected, fixed with 4% formaldehyde, and double stained with α-Me31B[43] and α-Dcp1[12] or α-Pcm[12].

Me31B is also required to recruit specific translational regulators to P-bodies in *Drosophila*. In cultured neurons, Me31B colocalizes with many conserved P-body and NG components including FMRP (Table 1)[12]. Here, we show that FMRP colocalizes to P-bodies with Me31B in wing imaginal discs (Fig. 3A–C) and S2 cells (Fig. 3D–F). The loss of Me31B inhibits the recruitment of FMRP to P-bodies (Fig. 3A–C). These results are interesting because of recent findings showing that: (a) FMRP and Me31B interact genetically and biochemically in *Drosophila*[12]; (b) FMRP colocalizes with Me31B and Tral, the *Drosophila* ortholog of RAP55/Scd6p, in granules in cleavage-stage embryos, and is required for normal Tral expression[69]; and (c) in the *Drosophila* germline, FMRP interacts with Orb, a homolog of the cytoplasmic polyadenylation binding protein, CPEB[70]. CPEB mediates many diverse processes including germ-cell development, cell division and cellular senescence, and synaptic plasticity[71].

Both Me31B/RCK and FMRP are core P-body components and translational repressors with important neuronal functions[12,72]. How are these functions (e.g., in dendritic branching) accomplished? One possibility is that Me31B and FMRP regulate neuronal processes by regulating miRNA and siRNA pathways[12,56,62]. The existing data are consistent with Me31B/RCK function as a direct regulator of the miRNA pathway. Me31B/RCK not only associates with Ago1, a key component of the miRNA pathway, but its depletion in wing imaginal discs also results in increased expression of a GFP-reporter mRNA, repressed by the *bantam* miRNA *in vivo*[12]. Function for Me31B outside of the *bantam* pathway is confirmed by new data indicating that Me31B is similarly required for translation repression mediated by a different miRNA, miR-2 (Fig. 4).

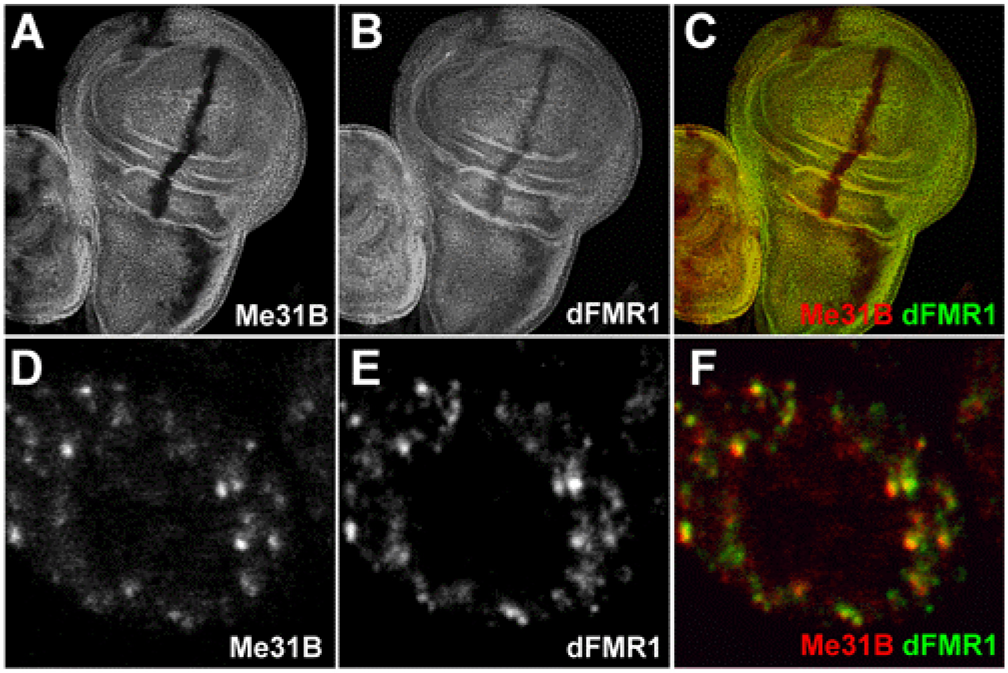

**FIGURE 3**. Depletion of Me31B reduces dFMR1 expression in wing imaginal discs, and Me31B and dFMR1 colocalize in P-bodies. (A–C) The loss of P-bodies in the wing imaginal disc by depletion of Me31B affects the recruitment of dFMR1 into foci. A Me31B hairpin construct[12] was expressed with patched-Gal4, reducing Me31B expression at the anterior/posterior boundary of the wing imaginal disc (marked by the absence of Me31B protein, A). For these experiments, wing discs of 3rd instar larvae were dissected, fixed with 4% formaldehyde and double stained with α-Me31B[43] and α-dFMR1 (5A11, Developmental Studies Hybridoma Bank). (D–F) In *Drosophila* Schneider, cells Me31B and dFMR1 colocalize in cytoplasmic foci, previously shown to be P-bodies (JH and MR, unpublished). For these experiments, *Drosophila* Schneider line 2 cells (S2) growing in M3 medium (Sigma) on glass cover slips were fixed with 4% Paraformaldehyde and counterstained with α-Me31B[43] and α-dFMR1 (5A11, Developmental Studies Hybridoma Bank).

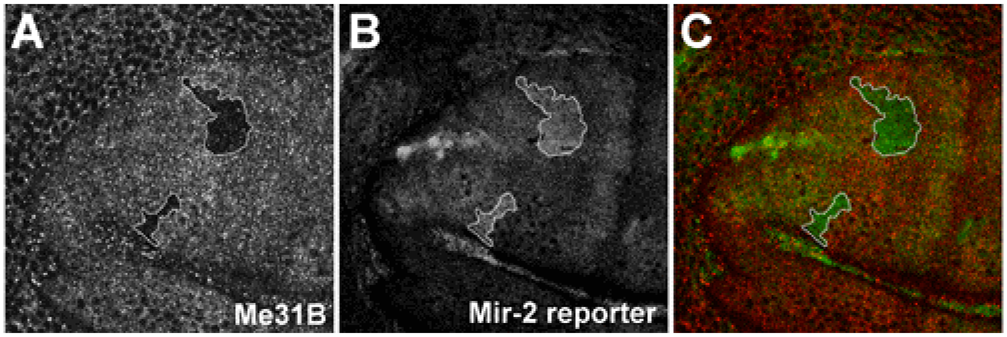

**FIGURE 4.** Me31B is required for the function of the endogenous microRNA mir-2. A mir-2 GFP-reporter[75] was used to measure the function of the endogenous miRNA mir-2. The up-regulation of GFP (B) in Me31B homozygous mutant clones (marked by the loss of Me31B expression, A) indicates that Me31B is required for the mir-2–dependent repression of GFP. In these experiments, mitotic recombination clones were induced by the FRT/FLP system, 48 ± 2 h after egg lay (AEL) by heat shock at 37ºC for 90 min; larval genotype used: *hsFLP; Me31B D1, FRT 40/FRT 40, arm-lacZ; mir2-reporter/+*. Wing discs were dissected 120 ± 2 h AEL, fixed with 4% formaldehyde and double stained with α-Me31B[43] and α-GFP (Molecular Probes).

## THE ROLE OF ME31B IN NEURONAL TRANSLATION

Experimental studies of the conserved DEAD-box helicase Me31B/RCK link the molecular control of miRNA function with specific subcellular sites (P-bodies), and the cellular process of neuronal plasticity. To summarize, Me31B/RCK is required for: (a) P-body formation (Fig. 2); (b) miRNA function (Fig. 4); and (c) sensory neuron dendrite morphogenesis, a process regulated by translational repressor proteins[12]. Despite the excitement this model induces, it is important to note that several important issues need to be addressed before a definite role for the Me31B in the synaptic regulation of locally stored miRNA target mRNAs can be concluded.

First, it remains possible that Me31B functions in global translational control, and that its synaptic phenotypes do not reflect a role in the miRNA pathway or in local translation. Given that the unicellular yeast, which lacks a miRNA pathway, requires the Me31B ortholog Dhh1p for translational control, further experiments are essential. These should, at the very least, ask whether Me31B regulates the translation of synapse-localized mRNAs. New technologies need to be developed or implemented to establish a direct role in local translation.

The mechanism(s) by which Me31B/RCK/Dhh1p functions in translation is also unclear. The interaction of Me31B/RCK/Dhh1p with numerous translational regulators like decapping enzymes, FMRP, RISC components, or CPEB[12,25,40,62,73] suggests a model where Me31B/RCK/Dhh1p recruits core P-body components and translational repressors, and mediates the aggregation of mRNPS into macroscopic mRNP particles, like P-bodies. Additionally, structural analysis of Dhh1p illustrates the potential RNA binding capability[74], substantiating the role of Me31B/RCK/Dhh1p as a general translational repressor. However, to get further insight into the functional mechanisms of Me31B/RCK/Dhh1p more detailed investigations are required.

In *Drosophila*, Me31B is required to recruit core components of P-bodies (Fig. 2) and NGs (Fig. 3) to macroscopic P-bodies in wing imaginal disc cells. The requirement of Me31B to recruit FMRP to P-bodies is of particular interest given that FMRP is present on NGs, interacts with RISC components, and acts as a regulator of synaptic plasticity[12,54,55,57,58]. How does Me31B's role in neuronal translational control derive from its interactions with FMRP, Tral, and other associated proteins? Me31B-repressed mRNAs can be derepressed and translated. To understand how repression is achieved and regulated, the identification of the interaction of Me31B with other proteins is important and must be interpreted in the context of dynamic and regulatable functions *in vivo*.

Another issue that remains unclear is the precise relationship between the accumulation of macroscopic P-bodies and miRNA function. Recent studies in *Drosophila* cell culture suggest that the regulation of miRNA function by P-body components does not require the formation of large P-bodies[19]. But what is the size of a functional P-body? The simplest model is that there is a pool of microscopic (not detectable by light microscopy) P-bodies that are sufficient for a basal level of P-body function. Macroscopic (large) P-bodies may have additional functions that are independent of mRNA decay and general- and miRNA-mediated translational repression pathways[8]. In a neuronal context, it will be important to understand how these observations relate to macroscopic NGs. Are large NGs solely involved in transport? Understanding NG formation and particle dynamics will likely provide significant insight into the mechanisms underlying synaptic mRNA translation.

Finally, and most importantly, we believe that major mechanistic insight into miRNA-mediated regulation of plasticity at the synapse will spring from the discovery that P-body components localize to Staufen- and FMRP-containing NGs[12]. Me31B, a shared component of P-bodies and NGs, is required for miRNA-mediated translational repression outside the nervous system, and ongoing experiments are designed to test the obvious hypothesis that Me31B mediates similar functions in neurons. Like Me31B, other core P-body components, including Dcp1/Dcp2 and GW182, appear to be intimately involved in translational regulation via the miRNA pathway. Thus, it is highly likely that more P-body components are involved.

## CONCLUDING THOUGHTS

Localization of P-bodies to neurites in *Drosophila* cell culture suggests that P-body formation and function may play a critical role in regulating, either directly or indirectly, local translation at the synapse – a mechanism widely held as essential for protein synthesis-dependent forms of LTM. To test and expand on this clearly articulated and increasingly well-supported hypothesis, future experiments need to address five broad questions. First, do P-body components regulate the translation of neuronal mRNAs? Second, do P-body components regulate the function of neuronal miRNAs? Third, are P-body components directly required (via a general- and/or miRNA-dependant mechanism) for regulation of local translation at the synapse? Fourth, is synaptic RNA turnover regulated by neuronal activity? Finally, are P-body components required for the formation of long-term memory?

## ACKNOWLEDGMENTS

This work was supported by a postdoctoral NRSA from the National Institute of Health (MH074262) to SAB, generous support to MR from the Science Foundation of Ireland, and the National Institute of Drug Abuse. We thank Akira Nakamura for his advice and generous help with analysis of Me31B. Special thanks are due to Roy Parker for sharing his remarkable insights into P-bodies and mRNA regulation.
